# Accounting for health expenditures, migration, and CO₂ emissions in the EU: a bidirectional analysis

**DOI:** 10.3389/fpubh.2026.1822318

**Published:** 2026-05-07

**Authors:** Melissa N. Cagle, Yasin G. Gençer, Mahmut Ünsa Şaşmaz, Ahmet Özen

**Affiliations:** 1 Department of Business Administration, Dokuz Eylü University, İzmir, Türkiye; 2 Department of International Trade and Finance, Yalova University, Yalova, Türkiye; 3 Department of Public Finance, Uşak University, Uşak, Türkiye; 4 Department of Public Finance, Dokuz Eylü University, İzmir, Türkiye

**Keywords:** bidirectional causality, CO₂ emissions, environmental health, European Union, health expenditure, net migration, panel data, panel Granger causality

## Abstract

**Background:**

Migration, health expenditures, and carbon emissions are critical policy concerns in the European Union. However, limited evidence exists on how these factors interrelate across diverse member states, and most existing studies examine only one pairwise linkage at a time, rely on non-EU settings, or employ methods that do not accommodate cross-sectional dependence and heterogeneous country dynamics. This study tests for bidirectional Granger-causal relationships among all three variable pairs across the EU-27.

**Methods:**

We compiled national-level data from 27 EU member states spanning 2000–2020. Using the Emirmahmutoğlu and Köse heterogeneous panel Granger causality test, which accommodates mixed integration orders and country-specific lag structures, with bootstrap critical values to address cross-sectional dependence, we tested for pairwise bidirectional predictive relationships among CO₂ (Carbon dioxide) emissions per capita, current health expenditure per capita, and net migration at both the panel and country levels. A Cross-Sectionally Augmented Autoregressive Distributed Lag (CS-ARDL) model was estimated as a robustness check to assess the long-run magnitude and sign of the identified relationships.

**Results:**

At the panel level, the Fisher test statistics reject the null of no Granger causality in both directions for all three variable pairs, CO₂ and net migration, health expenditure and net migration, and health expenditure and CO₂ (all *p* < 0.01), confirming panel-level bidirectional Granger causality. At the country level, however, the patterns are markedly heterogeneous: only Italy exhibits bidirectional causality between CO₂ and migration; four countries (Germany, Sweden, Croatia, Poland) show bidirectional health expenditure–CO₂ feedback; and Portugal and Slovenia show bidirectional migration–health expenditure linkages. Unidirectional results emerge in a further 17 countries, while Ireland, Luxembourg, and the Netherlands show no significant linkages. The CS-ARDL robustness analysis, estimated for the CO₂ equation only, confirms a significant negative long-run association between health expenditure and CO₂ and a significant positive long-run association between net migration and CO₂. Structural interpretation of the country-level heterogeneity identifies four broad regime types, integrated nexus countries with feedback dynamics, environment-sensitive migration regimes, demographic-pressure-driven systems, and structurally decoupled systems, though several countries exhibit multi-linkage profiles that span more than one category.

**Conclusion:**

The EU-wide panel results mask several distinct national pathways shaped by differences in energy mix, health-system design, and migration exposure. Policy responses should be regime-specific: decarbonising migrant-absorbing infrastructure in demographic-pressure systems, coupling pollution remediation with place-based investment in environment-sensitive regimes, strengthening migrant-inclusive healthcare in integrated nexus countries, and pairing pollution control with healthcare-sector decarbonisation in feedback systems.

## 1 Introduction

Across the European Union, policymakers face three pressures that are rarely examined together: rising and increasingly diverse migration flows, binding commitments to reduce greenhouse gas emissions, and growing strains on nationally distinct health systems. Each of these challenges has generated its own body of research and its own policy response, yet in practice they do not operate in isolation. Migration reshapes the populations that health systems must serve and the consumption patterns that drive emissions, while environmental degradation and health-system conditions may themselves influence where people move. These linkages are mediated by shared mechanisms, including urbanisation (1), shifts in energy demand (1, 2), and changes in the demographic composition of destination populations (3), that cut across all three policy domains. Understanding how these forces interact is not only an academic question but a governance one, with direct implications for how the EU balances free movement, health-system sustainability, and its climate targets.

Existing research offers partial insights into each of these channels. Studies on migration’s economic effects generally find that labour-market and public finance are more modest than commonly perceived, though outcomes vary by skill composition and institutional context (4). At the same time, evidence on the CO₂–health expenditure nexus suggests that environmental degradation may drive up healthcare costs, and that countries risk escalating health expenditure pressures if economic growth strategies are not aligned with environmental policy (5). Less is known about how these dynamics interact when migration is added to the picture.

Despite this, evidence on their interconnected dynamics within the EU remains limited. Existing research has focused largely on non-EU settings, especially the United States and China (4, 6), and has typically examined only one directional channel at a time, for example how migration affects emissions or how migrant populations affect healthcare utilisation (6). Moreover, the methods employed in these studies, ranging from static panel regressions that assume homogeneous effects across countries (6) to first-generation panel techniques that do not account for cross-sectional dependence (7, 8) and time-series approaches confined to individual countries (9), have generally not been designed to test for bidirectional Granger-causal relationships in heterogeneous multi-country panels. Much less is known about whether these three variables are linked bidirectionally across EU member states, despite the EU’s unique combination of open internal borders, binding emissions targets, and heterogeneous national health systems. The EU-27 is particularly suited to this analysis for reasons that distinguish it from alternative country groupings. Unlike the OECD or G20, whose members do not share open internal borders or binding collective emissions targets, EU member states operate under a common free-movement framework and the legally binding targets set out in the European Climate Law, including climate neutrality by 2050 and a net greenhouse gas reduction target of at least 55% by 2030 (10). At the same time, they differ markedly in migration exposure, energy mix, and health-system organisation, a combination of common institutional frameworks and structural heterogeneity across member states that is well suited to the heterogeneous panel Granger causality framework employed in this study. Moreover, the dominance of single-country designs (especially for the United States and China) in the existing literature leaves the cross-country dynamics that characterise the EU largely unexamined.

This study addresses that gap using a panel of 27 EU countries over the period 2000–2020. A panel framework is well suited to this setting because EU member states share common economic and policy shocks yet differ markedly in migration exposure (4), energy mix (11), and health-system structure (12). The Emirmahmutoğlu and Köse (13) panel Granger causality test is applied to net migration, current health expenditure per capita (hereafter HEXP), and CO₂ emissions, because it accommodates heterogeneous country dynamics and mixed integration orders; given the detected cross-sectional dependence, panel-level inference is based on bootstrap critical values. A CS-ARDL model is estimated as a supplementary robustness analysis to assess the long-run magnitude and sign of the identified relationships. HEXP denotes current health expenditure per capita and therefore includes public, private, and out-of-pocket health spending (14); accordingly, any fiscal implications discussed in this paper should be interpreted as indirect rather than as direct effects on government budget balances or convergence criteria.

The study uses net migration as its primary migration indicator. While net migration captures all population movements, including refugee flows, the analytical emphasis is on broader migration patterns rather than refugee-specific dynamics. The core research questions are: Does rising net migration place upward pressure on health expenditure in the EU, with implications for health system sustainability and resource planning? And could rising net migration contribute to higher carbon emissions, potentially hindering the EU’s goal of cutting net greenhouse gas emissions by 55% by 2030 (10)?

These questions carry immediate practical weight: member states absorbing large inflows may need to simultaneously scale up healthcare provision, staffing, infrastructure, and language-accessible services, while meeting emissions-reduction commitments that leave little room for the additional energy and resource demands such expansion entails.

Given the heterogeneity of EU member states in migration exposure, energy mix, and health-system organisation, we expect the Granger-causal relationships among these variables to differ across countries rather than follow a single EU-wide pattern, with bidirectional linkages emerging in some member states but not others.

To the best of our knowledge, this is the first study to test bidirectional Granger-causal relationships among all three variables, migration, health expenditure, and CO₂ emissions, within a single empirical framework for the EU-27, providing both country-specific and panel-level evidence on linkages that have previously been examined only in isolation or in non-EU settings. In doing so, the study contributes to environmental health economics by bridging three literatures that have largely developed in parallel, and to EU policy debate by identifying which member states exhibit predictive linkages among migration, emissions, and health spending, information that can inform the coordination of climate, health, and migration governance.

The results reveal panel-level bidirectional Granger causality for all three variable pairs, CO₂ and net migration, health expenditure and net migration, and health expenditure and CO₂ (all Fisher test statistics significant at the 1% level), but country-level patterns are markedly heterogeneous. Of the 27 member states, only seven exhibit bidirectional Granger causality for at least one variable pair, 17 show unidirectional linkages only, and three show no significant relationship in any direction. The CS-ARDL robustness analysis, which estimates the CO₂ equation, confirms a significant negative long-run association between health expenditure and CO₂ and a significant positive association between net migration and CO₂. Rather than treating this heterogeneity as unexplained, the paper interprets the country-level patterns through three structural conditioning mechanisms, differences in energy mix and pollution burden, health-system design and migrant coverage arrangements, and the scale of migration relative to carbon-intensive infrastructure, and proposes a broad four-category regime typology that links the statistical patterns to country-specific structural features, though several countries span more than one category. Policy recommendations are differentiated by regime type rather than applied uniformly across the EU-27, with each regime receiving targeted interventions grounded in the structural evidence developed in the discussion.

The remainder of this paper is organised as follows. Section 2 reviews the literature on the bidirectional causality among CO₂ emissions, health expenditure, and migration. Section 3 describes the data and econometric methodology. Section 4 reports the empirical results, including a country-level typology of Granger-causality profiles. Section 5 develops the structural interpretation of the heterogeneous results, introduces the regime typology, and discusses policy implications. Finally, the Conclusion summarises the findings, acknowledges limitations, and outlines directions for future research.

## 2 Literature review

The relationship between migration, carbon emissions, and health expenditure has been studied across several disciplines, environmental economics (11), health policy (15), and demography (16), but these strands of research have largely developed in parallel rather than in conversation with one another. This section reviews the evidence along three pairwise channels: CO₂ and health expenditure (Section 2.1), migration and CO₂ (Section 2.2), and migration and health expenditure (Section 2.3). Although each pair has its own empirical literature, the underlying mechanisms, demographic change (17), urbanisation (1, 11), consumption shifts (1, 18), and institutional capacity (12, 15), cut across all three, suggesting that in the EU context, where the 2015–2016 peak in arrivals underscored the interconnections between migration governance, resource allocation, and environmental policy (11), these relationships may be interlinked rather than operating as three separate phenomena.

Migration, in particular, connects the other two variables: a single sustained inflow can shift the pollution exposures that drive healthcare costs (19) and reshape the demographic profile on which per capita health expenditure depends (3). Before reviewing the pairwise evidence, it is important to clarify the macro-level constructs that anchor the empirical analysis. CO₂ emissions per capita serve as a proxy for country-level carbon intensity and environmental pressure (7, 11, 20), not as a measure of local pollution exposure or ambient air quality, constructs examined elsewhere using pollutants such as NO₂ or PM2.5 (6, 19, 21, 22). HEXP captures system-level health expenditure per capita, encompassing public, private, and out-of-pocket spending (15), and should not be conflated with healthcare quality, access, or individual-level service utilisation, which are the focus of a related but distinct body of work (23–26). Net Migration (MIGR) is the balance between population inflows and outflows, and is therefore distinct from immigrant stock or share (3, 21), gross inflows, or refugee-specific movements (9). Because much of the existing literature employs these related but non-identical measures, such as air pollutant concentrations (6, 19), foreign-born population shares (3, 21), or healthcare utilisation rates (23, 24, 26), the review that follows flags these measurement differences where they arise. Such studies provide relevant adjacent evidence, but they do not map directly onto the variables operationalised in this study; full measurement details and data sources are reported in Section 3.

### 2.1 The bidirectional relationship between CO₂ and HEXP

Research on CO₂ and health expenditure points to a potentially bidirectional relationship. Grossman’s (27) health capital model offers a useful lens: because environmental degradation, including pollution associated with CO₂-intensive energy use, accelerates the depreciation of health capital it raises the investment (i.e., expenditure) required to maintain a given health stock. Consistent with this logic, several studies find that higher emissions are associated with greater health burdens and healthcare costs (8, 20, 28). For example, Apergis et al. (20) found that higher per capita emissions across US states are associated with higher per capita health care expenditures, suggesting that emission reductions could yield tangible cost savings. In the other direction, expansion of health services may itself be energy-intensive, especially where demographic growth or rising service demand requires larger facilities and greater resource use (29). Chaabouni et al. (7) found that in low-income countries, a 1% increase in HEXP is associated with an approximately 0.2% increase in CO₂, though this relationship was not statistically significant for middle-income or global panels. In contrast, Li et al. (5) found that higher HEXP in countries such as China and India is negatively associated with CO₂, suggesting that the direction and sign of the HEXP–CO₂ relationship may differ across income groups.

The empirical evidence is not entirely uniform. Dritsaki & Dritsaki (30) found that rising greenhouse gas emissions are negatively associated with per capita health expenditure across G7 nations, though the magnitude varies considerably, from a 3.32% decline in Japan to only 0.28% in Italy per 1% increase in emissions. This suggests that institutional context, energy mix, and the structure of healthcare provision matter for how the CO₂–HEXP relationship unfolds.

These disparities may be compounded by migration: Ehler et al. (19) found that migrants in Germany are exposed to 16.4% more nitrogen dioxide and 1.9% more fine particulate matter than non-migrants, largely because they are overrepresented in polluted urban areas. Such differential exposure suggests that the CO₂–HEXP relationship may not affect all population groups equally, a consideration that becomes central when migration enters the picture.

Overall, the literature supports the possibility of bidirectional linkages between CO₂ and health expenditure, but most existing studies are based on non-EU settings or smaller country groups. Methodologically, these studies have employed a range of panel techniques, including panel ARDL (Autoregressive Distributed Lag) (8), VECM (Vector Error Correction Model) Granger causality (28), dynamic simultaneous equations (7), time-series ARDL (5), panel quantile regression (20), and FMOLS/DOLS (Fully Modified / Dynamic OLS) estimation (31), with varying attention to cross-sectional dependence and heterogeneous country dynamics, and not all formally test for bidirectional Granger causality (20, 30, 31). Wang et al. (32) applied a bootstrap ARDL approach to 18 OECD countries individually and found bidirectional short-run causality between health expenditure and CO₂ emissions in only New Zealand and Norway, with long-run cointegration detected in just three countries, underscoring the country-specific nature of these relationships; however, their time-series design does not incorporate migration and cannot exploit cross-country information within a panel framework. This leaves open the question of whether similar predictive relationships hold across the EU-27.

### 2.2 The bidirectional relationship between migration and CO₂

While the CO₂–HEXP relationship operates primarily through health-system costs and energy intensity, migration introduces a more direct demographic channel: by altering the size (17), location (16), and consumption patterns of populations (17, 18), it can reshape both emissions profiles (11) and health-system demands (3), simultaneously. Two theoretical frameworks anchor this relationship, the IPAT identity (17), which conceptualises environmental impact as a function of population size, per capita consumption, and technology (I = P × A × T), and (16) push-pull theory, which positions environmental conditions among the factors shaping migration decisions, but the empirical literature has developed these channels unevenly. Research on migration’s effect on emissions is far more developed than the reverse direction, and it is with this dominant channel that we begin.

Across studies on China, Europe, and the United States, migration is commonly associated with higher emissions through urbanisation, increased transport and housing demand, and shifts towards more carbon-intensive consumption in destination regions (1, 11, 18, 33, 34). For instance, Alola et al. (11) found a small but significant increase in CO₂ emissions associated with migration in France, Germany, and the United Kingdom. Scenario-based projections by Cafaro & Götmark (35), using population-differentiated emissions calculations for Germany, estimated that under a high-migration scenario, emissions would fall to only 88% of 2016 levels by 2050, compared with 56% under zero net migration. Liang et al. (18) estimated that the CO₂ footprint of international migrants reached 2.9 Gt in 2015, approximately 8% of global emissions, with roughly half of these flows directed from developing to developed countries. These findings are consistent with the IPAT logic: even where migrants’ per capita emissions are lower than those of the native-born population, the aggregate effect of population growth on total impact can outweigh per capita savings, particularly in already-industrialised economies where diminishing returns in resource efficiency are operative (17).

At the same time, the effect is context-dependent rather than uniform. Some studies show that migration can redistribute emissions spatially rather than simply raise them everywhere, while others suggest that technological change, cleaner energy adoption, or lower per-capita consumption among some migrant groups may partly offset population-pressure effects (21, 36–38).

The reverse direction, in which environmental conditions shape migration, has received less attention but is theoretically well grounded in push-pull theory (16). Available evidence suggests that environmental quality can shape migration decisions, though most studies measure local air pollutants rather than CO₂, the pollutants share common combustion sources and are therefore correlated with CO₂ at the country level (6). Climate-related shocks may also intensify human mobility by increasing livelihood insecurity and settlement pressures (39, 40). Lee (16) terms, rising CO₂ concentrations and their downstream consequences, air quality deterioration, heat stress, water scarcity, function as “minus” factors that can both push populations away from degraded origins and reduce the attractiveness of high-emission destinations, creating feedback loops between environmental change and population movement. As noted in Section 2, CO₂ emissions serve as a macro-level environmental proxy in this study and do not capture the full range of pollutants that may influence migration decisions.

Methodologically, the approaches used in this literature range from cross-sectional regressions that lack any temporal dimension (21) to static panel fixed-effects models that impose homogeneous slopes and do not test for stationarity or cross-sectional dependence (6), and panel studies that apply first-generation unit root and cointegration tests despite detecting cross-sectional dependence, with causality assessed only at the panel level (11). Taken together, the evidence implies a potentially bidirectional relationship between migration and emissions, theoretically anchored in the IPAT and push-pull frameworks, but it remains fragmented and largely non-EU in design, with existing studies typically examining either migration-induced emissions or environmental drivers of migration rather than testing both directions within a common empirical framework. This creates a practical policy tension: the same urban concentration that raises transport, housing, and energy demand also changes who is exposed to pollution and when that exposure translates into healthcare utilisation (11, 19), linking the migration–CO₂ channel directly to the health-expenditure pressures examined in the following subsection.

### 2.3 The bidirectional relationship between migration and HEXP

Whether migration raises or lowers health expenditure in destination countries is one of the most contested questions in this nexus (41), and the answer appears to depend on which migrants, at what stage of settlement, and under what institutional arrangements are being studied. Some studies argue that migrant inflows can increase healthcare demand, especially where legal or financial barriers delay treatment and shift utilisation towards emergency care (23, 24, 42, 43). Others find that migrants, particularly younger or newly arrived groups, use fewer services and may reduce per-capita health expenditure in the short run (3, 26, 41, 44). For instance, Bettin & Sacchi (3) found that a one percentage point increase in the immigrant share of the population in Italy was associated with a 3.8% reduction in per capita health spending, a pattern largely attributed to migrants’ younger demographic profile.

Grossman’s (27) health capital model helps reconcile these seemingly contradictory findings. The model treats health as a durable capital stock that depreciates over time and can be augmented through investment, including medical care, diet, and environmental conditions. Healthcare expenditure is not demanded for its own sake but is derived from the more fundamental demand for “good health” (27); the optimal stock of health at any point depends on the individual’s depreciation rate, wage rate, and efficiency in producing health (27). Because migration alters each of these determinants, by exposing individuals to new environments, labour-market conditions, and healthcare institutions (4), the model provides a micro-foundation for expecting migration to affect health expenditure patterns in destination countries. An important distinction follows from this logic: the individual decision to seek care is conceptually distinct from aggregate HEXP (as defined in Section 2), which reflects system-level resource allocation rather than individual utilisation behaviour (3, 24). HEXP, as used in this study, captures the latter; changes in HEXP therefore need not map one-to-one onto changes in individual service use among any particular population group.

Through the concept of health capital depreciation, the model predicts a dynamic, time-dependent relationship rather than a static one. The healthy immigrant effect (45), which suggests that migrants often arrive younger and healthier than the native-born population, can be interpreted as reflecting a higher initial health stock and a lower depreciation rate at the point of arrival, reducing the derived demand for medical care in the short run. However, this advantage may erode over time as depreciation rises through ageing, acculturation, occupational hazard exposure, and persistent barriers to routine care, implying that expenditure effects may shift in the longer run (41, 46). This health advantage is not uniform across all immigrant categories; Lu & Ng (47) found that while the effect persists among family-class immigrants, it is substantially weaker among refugees, a group that may require additional health monitoring and thus exert greater pressure on healthcare expenditures over time.

The reverse direction, whether health expenditure or health-system conditions influence migration, is less developed in the literature. Some studies suggest that access to healthcare and broader service provision can form part of destination attractiveness, particularly in rural–urban settings (48). Within Lee (16) push-pull framework, healthcare access and service quality can function as pull factors at destination, making regions with stronger health systems more attractive to potential migrants, particularly those facing inadequate provision at origin. However, healthcare expenditure itself is rarely treated as a primary driver of migration, which is more commonly explained by labour-market, family, political, and security factors (49, 50).

Methodologically, the few studies that incorporate a migration or refugee variable into the health expenditure nexus have relied on single-country time-series designs (9) or on panel simultaneous-equation models that impose homogeneous coefficients across units and infer causal direction from coefficient significance rather than from formal Granger-causality testing (48). Neither approach accounts for cross-sectional dependence or allows for heterogeneous dynamics across countries, limitations that the authors of Ahmad et al. (48) themselves acknowledge. The migration–HEXP relationship thus appears theoretically plausible in both directions, grounded in the logic of health capital accumulation and depreciation, but the evidence is mixed and often indirect, and rarely tested within an EU-wide panel framework that formally addresses these econometric constraints.

### 2.4 Research gap and hypotheses

What emerges from all three literatures is that migration, CO₂ emissions, and health expenditure should not be treated as isolated pairwise relationships. In the EU context, where free movement of persons coexists with binding emissions targets and nationally distinct health systems, these variables may be systematically linked: population movements can affect both environmental and health-system pressures, while environmental degradation and health-system conditions may in turn shape migration dynamics. In operational terms, migration simultaneously alters aggregate resource use, raising energy and infrastructure demand, changes who is exposed to environmental hazards, and reshapes the volume and timing of healthcare utilisation; these overlapping channels mean that isolating any single variable pair risks misattributing effects that flow through the other two. Recognising this broader interdependence motivates a comprehensive pairwise analysis that tests all three variable pairs within a single study using a common methodology.

Three gaps remain in the literature.

First, most studies examine only one pairwise linkage rather than the broader migration–emissions–health expenditure nexus. Second, the existing evidence is dominated by non-EU settings, especially the United States and China, leaving uncertainty about whether similar relationships hold across EU member states. Third, even when associations are identified, studies often focus on one direction only, leaving the bidirectional nature of these relationships insufficiently tested. These substantive gaps are compounded by methodological limitations in the existing empirical literature. Many of the panel studies examining the CO₂–health expenditure, migration–CO₂, and migration–health expenditure relationships rely on first-generation unit root and cointegration tests that assume cross-sectional independence (7, 8, 31), even though, where tested, cross-sectional dependence is consistently detected (11, 28). Several studies impose homogeneous slope coefficients across structurally diverse countries, whether through pooled mean group estimators that constrain long-run dynamics (8), system GMM with common coefficients within income or regional groups (7, 48), or static fixed-effects models that assume identical effects across all panel members (6). Where causality is formally tested, the methods used carry their own constraints. The VECM Granger causality approach (8, 28) retains long-run information through the error correction term but requires a prior cointegration step and cannot accommodate variables with mixed integration orders in levels. The Dumitrescu and Hurlin (51) procedure, when applied to differenced data, discards level information entirely and likewise cannot handle mixed integration orders (11, 20, 30). Other studies infer causal direction from the sign and significance of coefficients in simultaneous equations (7, 48) or static regressions (6, 21) whether migration, CO₂ emissions, and health expenditure are linked by bidirectional Granger-causal relationships across EU member states, and some do not test for causality at all (31, 52). Even the most methodologically careful studies in this literature, those that correctly diagnose cross-sectional dependence and heterogeneity and employ second-generation estimators for long-run coefficients, still rely on causality tests that produce only panel-level inference without country-specific results (20, 28, 30), or analyse individual countries in isolation without a common panel framework (5, 9). Taken together, these limitations mean that the question of whether migration, CO₂ emissions, and health expenditure are linked by bidirectional Granger-causal relationships across EU member states has not been tested with a method that simultaneously accounts for cross-sectional dependence, accommodates heterogeneous lag structures and mixed integration orders, and delivers both country-specific and panel-level Granger-causality inference, the approach adopted in the present study. To address these gaps, the present study tests the following directional hypotheses for the EU-27.

**Figure 1:**
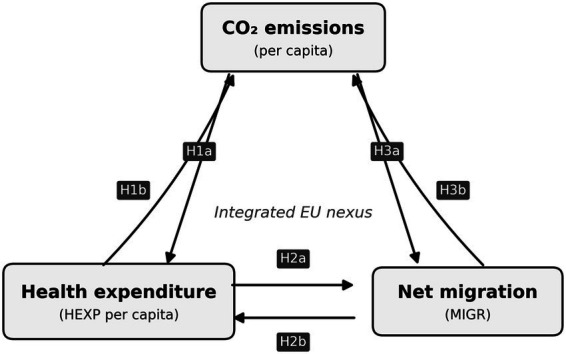
Conceptual framework of the bidirectional nexus among CO₂ emissions, health expenditure, and migration in the EU. It should be noted that the pairwise specification tests each variable pair separately rather than within a single trivariate system. This design choice is motivated by the degrees-of-freedom constraints noted above, but it means that the results identify pairwise predictive relationships rather than a fully simultaneous three-way interaction. The term “nexus” is used throughout this paper to denote the set of pairwise linkages among the three variables, not a jointly estimated trivariate causal system. Arrows represent hypothesised Granger-causal directions rather than structural causal effects: H1a CO₂ → HEXP; H1b HEXP → CO₂; H2a HEXP → MIGR; H2b MIGR → HEXP; H3a CO₂ → MIGR; H3b MIGR → CO₂.

Figure 1 summarises the study’s conceptual framework. It presents the EU nexus among the three variables and the six directional hypotheses tested in the panel Granger causality analysis.

*Hypothesis* 1a: CO₂ → HEXP.

*H*₀: CO₂ does not Granger-cause HEXP in the 27 EU countries.

*H*₁: CO₂ Granger-causes HEXP in at least one EU country.

*Hypothesis* 1b: HEXP → CO₂.

*H*₀: HEXP does not Granger-cause CO₂ in the 27 EU countries.

*H*₁: HEXP Granger-causes CO₂ in at least one EU country.

*Hypothesis* 2a: HEXP → MIGR.

*H*₀: HEXP does not Granger-cause MIGR in the 27 EU countries.

*H*₁: HEXP Granger-causes MIGR in at least one EU country.

*Hypothesis* 2b: MIGR → HEXP.

*H*₀: MIGR does not Granger-cause HEXP in the 27 EU countries.

*H*₁: MIGR Granger-causes HEXP in at least one EU country.

*Hypothesis* 3a: CO₂ → MIGR.

*H*₀: CO₂ does not Granger-cause MIGR in the 27 EU countries.

*H*₁: CO₂ Granger-causes MIGR in at least one EU country.

*Hypothesis* 3b: MIGR → CO₂.

*H*₀: MIGR does not Granger-cause CO₂ in the 27 EU countries.

*H*₁: MIGR Granger-causes CO₂ in at least one EU country.

Bidirectional Granger causality is inferred only when both directional null hypotheses for a given variable pair are rejected.

## 3 Methodology

This section describes the data, the pre-estimation diagnostic tests, the heterogeneous panel Granger-causality procedure, and the supplementary CS-ARDL robustness analysis.

### 3.1 Data

The study employs a balanced panel of 27 EU member states over the period 2000–2020, yielding 567 country-year observations. All data were obtained from the World Bank’s World Development Indicators. Table 1 presents the variables, their definitions, units of measurement, and indicator codes.

**Table 1:** Variables and their definitions.

Variable	Abbreviation	Unit	Indicator Code
CO₂ emissions per capita	CO₂	Metric tons per capita	EN. ATM. CO2E. PC
Current health expenditure per capita	HEXP	Current US$	SH. XPD. CHEX. PC. CD
Net migration	MIGR	Persons(5-year estimates)	SM. POP. NETM

In the EU, immigration is defined as the action by which a person establishes their usual residence in the territory of an EU Member State for a period that is, or is expected to be, of at least 12 months, having previously been usually resident in another EU Member State or a third country (52). Refugees, as defined by the EU, are third-country nationals or stateless persons who, owing to a well-founded fear of being persecuted for reasons of race, religion, nationality, political opinion, or membership of a particular social group, are outside their country of nationality and are unable or unwilling to avail themselves of that country’s protection (Directive 2011/95/EU) (53).

HEXP refers to current health expenditure per capita and captures public, private, and out-of-pocket health spending. Health expenditure is denominated in current US dollars rather than purchasing power parity (PPP) terms; while PPP-adjusted measures facilitate cross-country comparison of spending levels, the present analysis examines within-country temporal dynamics through a VAR (Vector Autoregression) framework, where the key variation is over time rather than across countries.

Net migration (MIGR) is defined as the number of immigrants minus the number of emigrants, including both citizens and noncitizens, and can therefore take negative values for countries experiencing net population outflows. It should be noted that the World Bank’s net migration series (SM. POP. NETM) reports five-year cumulative estimates derived from the UN Population Division’s World Population Prospects, in which net migration is calculated as a residual, the difference between total population change and natural increase. Although the World Bank assigns values to individual years, each figure represents a five-year total. This interpolation introduces artificial smoothness that may mask genuine year-to-year variation and mechanically induces persistence in the series, which could inflate the apparent predictive power of lagged migration values in a Granger-causality framework. This represents a real limitation for annual inference and should be borne in mind when interpreting the MIGR results. However, this data source remains the standard in cross-country panel studies of migration (11, 48), and no alternative annual net migration series with EU-27 coverage is currently available. The bootstrap critical values used in the Emirmahmutoğlu and Köse (13) test provide some robustness to non-standard serial correlation structures, though they do not fully resolve the interpolation-induced persistence concern. No logarithmic transformation was applied to any of the variables. While log transformation is common in panel studies to reduce skewness and facilitate elasticity interpretation, net migration, which takes both positive and negative values, cannot be log-transformed without additional manipulation (e.g., inverse hyperbolic sine transformation). To maintain consistency across all three variables and avoid introducing asymmetric transformations, all variables enter the analysis in levels. The lag-augmented VAR framework underlying the Emirmahmutoğlu and Köse (13) causality test imposes no distributional or sign restrictions on the variables, making level-form estimation appropriate.

The EU-27 was selected because it is the only regional grouping that combines legally open internal borders, binding collective emissions-reduction targets, and nationally distinct health systems, features absent from alternative panels such as the OECD or G20. The study period 2000–2020 is determined by the intersection of the three series: current health expenditure data under the WHO’s System of Health Accounts 2011 framework begin in 2000, while the CO₂ emissions per capita series (sourced from Climate Watch historical emissions via the World Bank) ends in 2020. This 21-year window also captures several substantively important developments, including the 2004 and 2007 EU enlargements, the 2008 global financial crisis, the 2015–2016 migration peak, and the onset of the COVID-19 pandemic, whose effects on the variables of interest are absorbed within the country-specific VAR dynamics estimated for each member state.

This study examines pairwise Granger-causal relationships among CO₂, HEXP, and MIGR in both directions for each variable pair. The analysis employs a pairwise specification across three variables. Each variable pair is estimated as a separate bivariate VAR; adding further variables to any equation would rapidly consume degrees of freedom given the limited time dimension relative to the cross-sectional dimension, increasing the risk of overfitting. While the omission of additional variables may in principle affect Granger causality inference (54), and in a pairwise design, each equation excludes the remaining study variable, so results are unconditional on it, the specification was chosen because expanding to higher-dimensional VARs with the present time dimension would further reduce degrees of freedom and increase the risk of overfitting. The pairwise results should therefore be interpreted as unconditional predictive relationships rather than as effects net of other variables in the study. The use of a heterogeneous panel causality approach for EU health expenditure data is further supported by Şentürk et al. (55), who applied the Kónya (56) bootstrap causality test to health expenditures and sustainable development in 11 new EU member states over 2000–2020, finding that causal interactions differ significantly across countries.

### 3.2 Estimation strategy

To select the appropriate econometric procedures, the data were subjected to a sequential battery of pre-estimation diagnostic tests. The sequence follows a decision-tree logic: cross-sectional dependence (CSD) testing determines whether first- or second-generation methods are appropriate; slope homogeneity testing determines whether heterogeneous or homogeneous estimators should be used; and unit root testing establishes the integration properties of the series, which in turn determine the lag augmentation parameter (dmax) required for the Granger causality test.

Cross-sectional dependence among the variables is tested first, as its presence can severely bias panel estimation results and invalidate first-generation econometric procedures Pesaran (57). The null hypothesis is cross-sectional independence, that is, the error terms across countries are uncorrelated (H₀: Cov(ε_it_, ε_jt_) = 0 for all i ≠ j). Three tests are employed: the Breusch–Pagan LM (Lagrange Multiplier) test (58), which is appropriate when T is large relative to N; Pesaran (57) CD test, which performs well across a wide range of T and N dimensions; and the bias-adjusted LM test Pesaran et al. (59), which corrects the size distortions of the Breusch–Pagan LM test when N is large relative to T. Given the panel dimensions of this study (*T* = 21, *N* = 27), where N slightly exceeds T, the Pesaran CD test is the most reliable of the three, as the Breusch–Pagan LM statistic is known to exhibit size distortions in panels where N is not small relative to Pesaran (57). All three tests are nonetheless reported for completeness. A *p*-value below 0.05 leads to rejection of the null hypothesis of cross-sectional independence.

Slope homogeneity is then examined using the Pesaran & Yamagata (60) delta test, which tests whether the slope coefficients are identical across all panel members (H₀: *β*ᵢ = β for all i) against the alternative that slopes differ across a non-zero fraction of countries. Both the delta tilde (Δ˜) and its small-sample adjusted version (Δ˜_adj) are reported, the latter being particularly relevant given the moderate dimensions of the present panel. If slope homogeneity is rejected, this implies that the relationships among the variables differ across EU member states, ruling out homogeneous panel causality tests and supporting the use of the Emirmahmutoğlu and Köse (13) procedure, which allows for country-specific heterogeneity in both lag structures and causal relationships.

If cross-sectional dependence is confirmed, first-generation panel unit root tests, such as the Levin et al. (61) and Im et al. (62) tests, are inappropriate, as they assume cross-sectional independence and suffer from severe size distortions when this assumption is violated. Accordingly, stationarity is assessed using the Pesaran (63) CADF/CIPS (Cross-sectionally Augmented IPS / Dickey-Fuller) second-generation panel unit root test, which accounts for cross-sectional dependence by augmenting the standard ADF regression with cross-sectional averages of the lagged level and first difference of the series. The null hypothesis is that the series contains a unit root (H₀: non-stationary) against the alternative of stationarity. The CADF statistic is computed for each cross-sectional unit, and the CIPS statistic is obtained as the simple average of the individual CADF statistics across all countries Pesaran (63). Critical values are drawn from the simulation-based tables in Pesaran (63) rather than from standard distributions. The unit root results serve a dual purpose: they establish the stationarity properties of each variable and determine the maximum order of integration (dmax), which is used to augment the VAR lag structure in the subsequent Emirmahmutoğlu and Köse (13) causality test.

### 3.3 Emirmahmutoğlu and Köse panel Granger causality test

Granger-causal relationships are tested using the Emirmahmutoğlu and Köse (13) panel Granger causality procedure, which extends the Toda & Yamamoto (64) lag-augmented VAR (LA-VAR) approach to heterogeneous panel settings. The Toda & Yamamoto (64) approach resolves a well-known problem in causality testing: when variables are integrated or cointegrated, standard Wald tests for Granger non-causality have non-standard asymptotic distributions, rendering conventional critical values unreliable. Toda & Yamamoto (64) address this by intentionally overfitting the VAR with dmax additional lags, where dmax is the maximum order of integration among the variables, and then testing restrictions only on the first k coefficient matrices. Because these coefficients retain standard asymptotic normality regardless of the integration properties of the system, the Modified Wald (MWALD) statistic follows a conventional χ^2^(k) distribution under the null hypothesis of non-causality Toda & Yamamoto (64). This approach has the important advantage of allowing Granger causality testing without requiring all variables to share the same integration order or requiring a separate cointegration step Westerlund & Edgerton (65) before testing, making it suitable for panels with mixed I(0) and I(1) variables.

Emirmahmutoğlu and Köse (13) extend this framework to heterogeneous panels by estimating a separate LA-VAR for each cross-sectional unit, allowing both lag structures and causal relationships to differ across countries. For each country i, a VAR of order (kᵢ + dmaxᵢ) is estimated in levels by OLS, where kᵢ is the country-specific optimal lag length and dmaxᵢ is the maximum order of integration. In the present study, the country-specific lag length kᵢ was selected using the Akaike Information Criterion (AIC) with a maximum lag order of 3, given that the limited time dimension precludes higher-order specifications without excessive loss of degrees of freedom. Based on the CIPS unit root results reported in Section 4, dmaxᵢ was set to 1 for all countries.

The three bivariate models estimated in this study are specified as follows:

Model 1: CO₂ and HEXP.


$$\begin{array}{c} \mathrm{CO}{₂}_{\mathrm{it}}=\alpha {₁}_{i}+{\Sigma_{j=₁}}^{\mathrm{ki}+d\max i}\;\beta ₁{₁}_{\mathrm{ij}}\;\mathrm{CO}{₂}_{i,t-j}\\ +{\Sigma_{j=₁}}^{\mathrm{ki}+d\max i}\;\beta ₁{₂}_{\mathrm{ij}}\;{\text{HEXP}}_{i,t-j}+\varepsilon {₁}_{\mathrm{it}}. \end{array}$$

$$\begin{array}{c} {\text{HEXP}}_{\mathrm{it}}=\alpha {₂}_{i}+{\Sigma_{j=₁}}^{\mathrm{ki}+d\max i}\;\beta ₂{₁}_{\mathrm{ij}}\;{\text{HEXP}}_{i,t-j}\\ +{\Sigma_{j=₁}}^{\mathrm{ki}+d\max i}\;\beta ₂{₂}_{\mathrm{ij}}\;\mathrm{CO}{₂}_{i,t-j}+\varepsilon {₂}_{\mathrm{it}}. \end{array}$$

Model 2: CO₂ and MIGR.


$$\begin{array}{c} \mathrm{CO}{₂}_{\mathrm{it}}=\alpha {₃}_{i}+{\Sigma_{j=₁}}^{\mathrm{ki}+d\max i}\;\beta ₃{₁}_{\mathrm{ij}}\;\mathrm{CO}{₂}_{i,t-j}\\ +{\Sigma_{j=₁}}^{\mathrm{ki}+d\max i}\;\beta ₃{₂}_{\mathrm{ij}}\;{\text{MIGR}}_{i,t-j}+\varepsilon {₃}_{\mathrm{it}}. \end{array}$$

$$\begin{array}{c} {\text{MIGR}}_{\mathrm{it}}=\alpha {₄}_{i}+{\Sigma_{j=₁}}^{\mathrm{ki}+d\max i}\;\beta ₄1_{\mathrm{ij}}\;{\text{MIGR}}_{i,t-j}\\ +{\Sigma_{j=₁}}^{\mathrm{ki}+d\max i}\;\beta ₄{₂}_{\mathrm{ij}}\;\mathrm{CO}{₂}_{i,t-j}+\varepsilon {₄}_{\mathrm{it}}. \end{array}$$

Model 3: HEXP and MIGR.


$$\begin{array}{c} {\text{HEXP}}_{\mathrm{it}}=\alpha 5_{i}+{\Sigma_{j=₁}}^{\mathrm{ki}+d\max i}\;\beta 51_{\mathrm{ij}}\;{\text{HEXP}}_{i,t-j}\\ +{\Sigma_{j=₁}}^{\mathrm{ki}+d\max i}\;\beta 5{₂}_{\mathrm{ij}}\;{\text{MIGR}}_{i,t-j}+\varepsilon 5_{\mathrm{it}}. \end{array}$$

$$\begin{array}{c} {\text{MIGR}}_{\mathrm{it}}=\alpha 6_{i}+{\Sigma_{j=₁}}^{\mathrm{ki}+d\max i}\;\beta 61_{\mathrm{ij}}\;{\text{MIGR}}_{i,t-j}\\ +{\Sigma_{j=₁}}^{\mathrm{ki}+d\max i}\;\beta 6{₂}_{\mathrm{ij}}\;{\text{HEXP}}_{i,t-j}+\varepsilon 6_{\mathrm{it}}. \end{array}$$

Where i = 1, ., 27 (EU member states); t = 1, ., T (time periods); kᵢ = country-specific lag length selected by AIC (maximum = 3); dmaxᵢ = maximum order of integration (= 1 based on CIPS results); αᵢ = country-specific intercept; and ε_it_ = error term.

In each model, Granger causality from x to y is tested by a Wald test on the null hypothesis H₀: the coefficients on the first kᵢ lags of x in the y equation are jointly zero, while the additional dmax lags are left unrestricted to ensure the Wald statistic follows its standard asymptotic χ^2^(kᵢ) distribution under the null Toda & Yamamoto (64). Country-level Wald test statistics and *p*-values are obtained from the individual augmented VAR models, and panel-level inference is derived by combining the country-specific p-values using Fisher (66) meta-analytic procedure:


$$\lambda =-2\;{\Sigma_{i=1}}^{N}\;\ln(\pi_{i}).$$

where πᵢ is the p-value of the Wald statistic for country i. Under cross-sectional independence and the null hypothesis of non-causality for all countries, λ follows a χ^2^(2 N) distribution. However, given the cross-sectional dependence confirmed by the Pesaran (57) CD test (Table 2), the asymptotic χ^2^(2 N) distribution is invalid. Accordingly, bootstrap critical values, generated by resampling residual vectors across time while preserving the contemporaneous cross-sectional correlation structure, are used to obtain valid inference. All estimations were performed in EViews.

**Table 2:** Results of cross-sectional dependence and homogeneity.

Variable	CDLM1 Breusch & Pagan (58)		CDLM2 Pesaran (57)		CDLM Pesaran (57)		Bias-adjusted CD test	
	Test stat.	*p* value	Test stat.	*p* value	Test stat.	*p* value	Test stat.	*p* value
Panel A: Cross-sectional dependence tests								
CO₂	4079.484	0.0000	140.7225	0.0000	140.0475	0.0000	49.79034	0.0000
HEXP	5863.852	0.0000	208.0692	0.0000	207.3942	0.0000	75.66391	0.0000
MIGR	2634.407	0.0000	86.18166	0.0000	85.50666	0.0000	3.553051	0.0000
Panel B: Slope homogeneity tests								
Test	Test statistic	*p*-value	Note: The null hypothesis is slope homogeneity (i.e., homogeneous parameters across countries). Rejection indicates heterogeneous slopes, supporting the use of estimation methods that allow for country-specific parameters.					
Δ˜ (Delta tilde)	14.286	0.000						
Δ˜_adj (Adjusted Delta tilde)	16.142	0.000						

It is important to note that the alternative hypothesis in this framework is heterogeneous: rejection of the panel null does not imply that Granger causality holds in all 27 member states, but rather that it holds in a sufficient number of countries to reject the joint null. This is why both panel-level and country-specific results are reported in Tables 3–5. It should also be noted that the present panel dimensions (*T* = 21, *N* = 27) depart from the test’s preferred configuration of T > N; bootstrap inference provides a size correction that mitigates finite-sample distortions under these conditions Emirmahmutoğlu and Köse (13).

**Table 3:** Emirmahmutoğlu and Köse causality test results (CO₂ → MIGR, MIGR → CO₂).

Countries	CO₂ → MIGR		MIGR → CO₂	
	Statistics	Prob. value	Statistics	Prob. value
Austria	1.049	0.306	0.006	0.941
Belgium	1.362	0.506	2.259	0.323
France	5.623*	0.060	0.234	0.890
Germany	0.135	0.935	3.545	0.170
Italy	15.020***	0.002	10.035**	0.018
Luxembourg	0.129	0.720	0.952	0.329
Czechia	0.320	0.572	0.012	0.912
Estonia	5.219**	0.022	0.157	0.692
Finland	0.001	0.972	1.928	0.165
Hungary	0.523	0.470	3.512*	0.061
Lithuania	8.832**	0.032	3.877	0.275
Malta	0.945	0.815	16.399***	0.001
Portugal	0.191	0.662	1.978	0.160
Slovak Republic	8.981**	0.030	2.882	0.410
Sweden	10.991**	0.012	1.790	0.617
Slovenia	3.758	0.289	4.095	0.251
Spain	3.373	0.338	5.210	0.157
Latvia	8.525**	0.036	1.297	0.730
Ireland	5.214	0.157	4.480	0.214
Greece	7.295*	0.063	2.984	0.394
Denmark	0.411	0.814	10.533***	0.005
Croatia	0.723	0.868	20.105***	0.000
Bulgaria	1.603	0.449	2.730	0.255
Netherlands	3.916	0.141	0.577	0.749
Cyprus	4.045	0.132	5.054*	0.080
Poland	0.510	0.917	4.831	0.185
Romania	19.058***	0.000	2.781	0.249
Fisher test statistic	107.156***	0.000	104.003***	0.000

*** Denotes significance at the 1% level, ** denotes significance at the 5% level, and * denotes significance at the 10% level.

**Table 4:** Emirmahmutoğlu and Köse causality test results (HEXP → MIGR, MIGR → HEXP).

Countries	HEXP→ MIGR		MIGR → HEXP	
	Statistics	Prob. value	Statistics	Prob. value
Austria	5.382	0.146	8.184**	0.042
Belgium	5.358*	0.069	0.175	0.916
France	13.041***	0.005	0.589	0.899
Germany	4.914	0.178	3.356	0.340
Italy	2.318	0.509	6.618*	0.085
Luxembourg	1.870	0.600	3.721	0.293
Czechia	4.178	0.124	0.529	0.768
Estonia	3.644*	0.056	0.017	0.896
Finland	5.356*	0.069	0.089	0.956
Hungary	9.284**	0.026	5.698	0.127
Lithuania	0.521	0.914	0.054	0.997
Malta	7.295*	0.063	3.175	0.365
Portugal	6.615*	0.085	7.824**	0.050
Slovak Republic	7.842**	0.049	0.958	0.811
Sweden	4.147	0.246	20.038***	0.000
Slovenia	7.578*	0.056	12.951***	0.005
Spain	0.201	0.654	4.872**	0.027
Latvia	0.321	0.571	0.694	0.405
Ireland	0.126	0.939	3.727	0.155
Greece	0.314	0.855	1.999	0.368
Denmark	2.652	0.266	1.658	0.437
Croatia	5.733	0.125	0.627	0.890
Bulgaria	0.490	0.783	12.458***	0.002
Netherlands	2.105	0.349	1.154	0.562
Cyprus	0.054	0.817	2.768*	0.096
Poland	2.353	0.125	0.000	0.990
Romania	0.089	0.766	0.022	0.882
Fisher test statistic	90.565***	0.001	92.942***	0.001

*** Denotes significance at the 1% level, ** denotes significance at the 5% level, and * denotes significance at the 10% level.

**Table 5:** Emirmahmutoğlu and Köse causality test results (HEXP → CO₂, CO₂ → HEXP).

Countries	HEXP→ CO₂		CO₂ → HEXP	
	Statistics	Prob. value	Statistics	Prob. value
Austria	4.330	0.228	7.179*	0.066
Belgium	2.393	0.495	3.064	0.382
France	1.906	0.386	7.057**	0.029
Germany	5.996**	0.050	9.144**	0.010
Italy	1.547	0.214	1.878	0.171
Luxembourg	0.056	0.813	0.296	0.587
Czechia	3.517*	0.061	1.664	0.197
Estonia	16.396***	0.000	4.346	0.114
Finland	4.909**	0.027	2.106	0.147
Hungary	3.804	0.283	12.356***	0.006
Lithuania	0.375	0.541	9.666***	0.002
Malta	24.973***	0.000	3.257	0.354
Portugal	0.003	0.958	0.015	0.903
Slovak Republic	0.448	0.503	1.570	0.210
Sweden	5.311*	0.070	8.060**	0.018
Slovenia	2.808*	0.094	1.075	0.300
Spain	0.250	0.617	0.449	0.503
Latvia	0.495	0.482	2.017	0.156
Ireland	1.443	0.230	0.163	0.686
Greece	1.775	0.183	0.494	0.482
Denmark	3.754	0.289	0.776	0.855
Croatia	8.376**	0.039	19.483***	0.000
Bulgaria	0.025	0.874	0.015	0.901
Netherlands	2.406	0.121	1.041	0.308
Cyprus	0.106	0.745	0.231	0.631
Poland	5.730*	0.057	8.332**	0.016
Romania	6.063	0.109	4.632	0.201
Fisher test statistic	115.396***	0.000	117.682***	0.000

*** Denotes significance at the 1% level, ** denotes significance at the 5% level, and * denotes significance at the 10% level.

The Emirmahmutoğlu and Köse test offers several advantages over alternative panel Granger causality procedures for the present analysis. Unlike the Dumitrescu and Hurlin (51) test, which requires stationary variables and imposes a common lag length across all cross-sections, the Emirmahmutoğlu and Köse procedure accommodates mixed integration orders in levels and allows heterogeneous lag structures. The Kónya (56) SUR (Seemingly Unrelated Regressions)-based approach is inapplicable here, as it requires T to substantially exceed N. Moreover, unlike standard panel VAR methods that impose homogeneous slope coefficients, the Emirmahmutoğlu and Köse framework allows causal relationships to differ across countries, a feature supported by the rejection of slope homogeneity reported in Table 2.

It should be noted that Granger causality tests assess predictive precedence rather than structural causation. A finding that X Granger-causes Y indicates that past values of X contain statistically significant information for predicting Y, beyond what is contained in Y’s own past values. The causal interpretations offered in this study should be understood within this framework. Country-level results significant at the 10% level should be interpreted as providing suggestive rather than strong evidence of Granger causality, in contrast to findings significant at the 5% or 1% level.

### 3.4 CS-ARDL robustness analysis

To complement the directional findings from the Granger causality analysis with evidence on the long-run magnitude and sign of the identified relationships, a Chudik & Pesaran CS-ARDL model (67) is estimated with CO₂ emissions as the dependent variable and health expenditure and net migration as regressors. The pre-estimation diagnostics reported earlier in this section, including the Pesaran (57) CD test confirming cross-sectional dependence across all three variables (Table 2), the Pesaran & Yamagata (60) slope homogeneity test rejecting homogeneous parameters across countries (Table 2), and the Pesaran (63) CIPS unit root test indicating that all three variables are I(1) (Table 6), jointly motivate the choice of CS-ARDL, which accounts for both cross-sectional dependence and slope heterogeneity by augmenting the model with cross-sectional averages of the dependent and independent variables and their lags.

**Table 6:** Results of second generation Pesaran CIPS unit root test.

Variables	Constant			
	Level		First differences	
	t st.	Prob.	t st.	Prob.
CO₂	−1.93142	> = 0.10	−2.77799***	<0.01
HEXP	−1.50648	> = 0.10	−2.37495***	<0.01
MIGR	−1.76125	> = 0.10	−2.36592***	<0.01

*** denotes significance at the 1% level, ** denotes significance at the 5% level, and * denotes significance at the 10% level.

The model is estimated using the Mean Group (MG) estimator with an ARDL(1,1,1) specification, one lag for the dependent variable and one lag for each regressor, as the short time series constrains the lag structure to ARDL(1,1,1). The MG estimator allows for fully heterogeneous slope coefficients across countries, consistent with the rejection of slope homogeneity reported in Table 2. The truncation lag for the cross-sectional averages was set following the rule pT = ⌊T^(1/3)⌋ recommended by Chudik & Pesaran (67), yielding pT = 2 for the present sample. The error correction term (ECT) provides evidence on the speed of adjustment towards long-run equilibrium; a negative and statistically significant ECT confirms the existence of a stable long-run relationship between the variables, serving as implicit evidence of cointegration Chudik & Pesaran (67).

Because the primary objective of this study is to test for bidirectional predictive linkages using the Emirmahmutoğlu and Köse (13) framework rather than to estimate a full structural system, the CS-ARDL is estimated for the CO₂ equation only; full system estimation with all three dependent variables is left for future research. It should also be noted that the relatively short time dimension of the panel (T = 21) is below the sample sizes for which CS-ARDL has been shown to perform optimally in Monte Carlo simulations Chudik et al. (68); accordingly, the CS-ARDL results should be interpreted as supplementary evidence that complements, rather than substitutes for, the Granger causality findings.

## 4 Findings

This section reports the pre-estimation diagnostic results, the panel Granger causality results, and the CS-ARDL robustness results.

### 4.1 Pre-estimation diagnostics

Table 2 presents the cross-sectional dependence and slope homogeneity test results. The null of cross-sectional independence is rejected for CO₂, HEXP, and MIGR under all reported CSD statistics (all *p*-values < 0.001). Accordingly, tests that account for cross-sectional dependence were preferred in the subsequent analysis.

Both the delta tilde (Δ˜ = 14.286, *p* = 0.000) and adjusted delta tilde (Δ˜_adj = 16.142, *p* = 0.000) statistics reject the null hypothesis of slope homogeneity at the 1% significance level. This confirms that the relationships among the variables are heterogeneous across the 27 EU countries, further supporting the use of the Emirmahmutoğlu and Köse (13) causality test, which accommodates country-specific heterogeneity.

The results of the Pesaran (63) CIPS unit root test are presented in Table 6. The CIPS results indicate that all three series are non-stationary in levels. Following first differencing, the null hypothesis of a unit root is rejected for all three variables at the 1% significance level. Since all three variables are integrated of order one, the maximum order of integration (dmax) is set to 1 for all countries in the subsequent causality analysis.

### 4.2 Panel Granger causality results

Tables 3–5 report the panel and country-specific Granger causality results for the three variable pairs. Table 3 reports the results for CO₂ and MIGR. At the panel level, the Fisher test statistic rejects the null of no Granger causality in both directions (CO₂ → MIGR: *λ* = 107.156, *p* < 0.01; MIGR → CO₂: λ = 104.003, *p* < 0.01), indicating bidirectional predictive linkages between CO₂ and net migration. At the country level, CO₂ → MIGR is significant in nine member states, France, Estonia, Lithuania, the Slovak Republic, Sweden, Latvia, Greece, Romania, and Italy, whereas MIGR → CO₂ is significant in six, Hungary, Malta, Denmark, Croatia, Cyprus, and Italy. Italy is the only country exhibiting bidirectional Granger causality for this variable pair. The remaining 13 member states show no significant Granger-causal relationship in either direction.

Table 4 reports the results for HEXP and MIGR. The Fisher test statistic rejects the null in both directions (HEXP → MIGR: *λ* = 90.565, *p* < 0.01; MIGR → HEXP: λ = 92.942, *p* < 0.01), indicating panel-level bidirectional Granger causality. At the country level, HEXP → MIGR is significant in nine member states, Belgium, France, Estonia, Finland, Hungary, Malta, the Slovak Republic, Portugal, and Slovenia, whereas MIGR → HEXP is significant in eight, Austria, Italy, Sweden, Spain, Bulgaria, Cyprus, Portugal, and Slovenia. Portugal and Slovenia exhibit significance in both directions, making them the only countries with bidirectional Granger causality for this variable pair. Twelve member states show no significant relationship in either direction.

Table 5 reports the results for HEXP and CO₂. The Fisher test statistic rejects the null in both directions (HEXP → CO₂: λ = 115.396, *p* < 0.01; CO₂ → HEXP: λ = 117.682, *p* < 0.01), indicating panel-level bidirectional Granger causality. At the country level, HEXP → CO₂ is significant in nine member states, Czechia, Estonia, Finland, Malta, Slovenia, Germany, Sweden, Croatia, and Poland, whereas CO₂ → HEXP is significant in eight, Austria, France, Hungary, Lithuania, Germany, Sweden, Croatia, and Poland. Germany, Sweden, Croatia, and Poland exhibit significance in both directions, making them the four countries with bidirectional Granger causality for this variable pair. Fourteen member states show no significant relationship in either direction.

These country-level results reveal substantial heterogeneity across the EU-27. Bidirectional Granger causality emerges in a small number of member states for each variable pair, Italy for CO₂ and MIGR, Portugal and Slovenia for HEXP and MIGR, and Germany, Sweden, Croatia, and Poland for HEXP and CO₂, while the majority of countries exhibit either unidirectional or no significant relationships. This pattern is consistent with the rejection of slope homogeneity in Table 2 and underscores the importance of reporting country-specific results alongside panel-level inference. Indeed, the large number of distinct country-level profiles, ranging from countries with no significant linkages in any direction to those with significant results across multiple variable pairs (e.g., Sweden, which exhibits unidirectional CO₂ → MIGR, unidirectional MIGR → HEXP, and bidirectional HEXP ↔ CO₂), suggests that the pairwise Granger-causality patterns vary substantially across member states. This heterogeneity reinforces the methodological rationale for the Emirmahmutoğlu and Köse (13) framework, which allows causal relationships, lag structures, and significance patterns to vary freely across the panel, as developed in Section 5.

### 4.3 CS-ARDL robustness results

Table 7 reports the CS-ARDL estimation results with CO₂ emissions as the dependent variable. In the long run, health expenditure is negatively and statistically significantly associated with CO₂ emissions (coefficient = −0.425, *p* < 0.01), while net migration is positively and significantly associated with CO₂ (coefficient = 0.158, *p* < 0.05). In the short run, the effects are more limited: the first-differenced health expenditure coefficient is negative but not statistically significant (*p* = 0.231), whereas the first-differenced migration coefficient is positive and marginally significant (*p* = 0.059). The error correction term (ECT = −0.340, *p* < 0.01) is negative and statistically significant, confirming the existence of a long-run equilibrium relationship. The magnitude of the ECT implies that approximately 34% of any short-run deviation from equilibrium is corrected in each period, corresponding to a return to long-run equilibrium in roughly three periods.

**Table 7:** CS-ARDL estimation results (dependent variable: CO2).

Variables	Coefficient	Std. Error	t-Statistic	*p*-value
Long-run dynamics				
Health (Health Expenditures)	−0.425***	0.103	−4.12	0.000
Migration	0.158**	0.064	2.45	0.015
Short-run dynamics				
Δ Health	−0.112	0.093	−1.20	0.231
Δ Migration	0.085*	0.045	1.89	0.059
Error correction				
ECT (−1)	−0.340***	0.065	−5.22	0.000

***, **, and * denote statistical significance at the 1, 5, and 10% levels, respectively. Δ represents the first-difference operator.

### 4.4 Country-level heterogeneity and causality profiles

**Figure 2:**
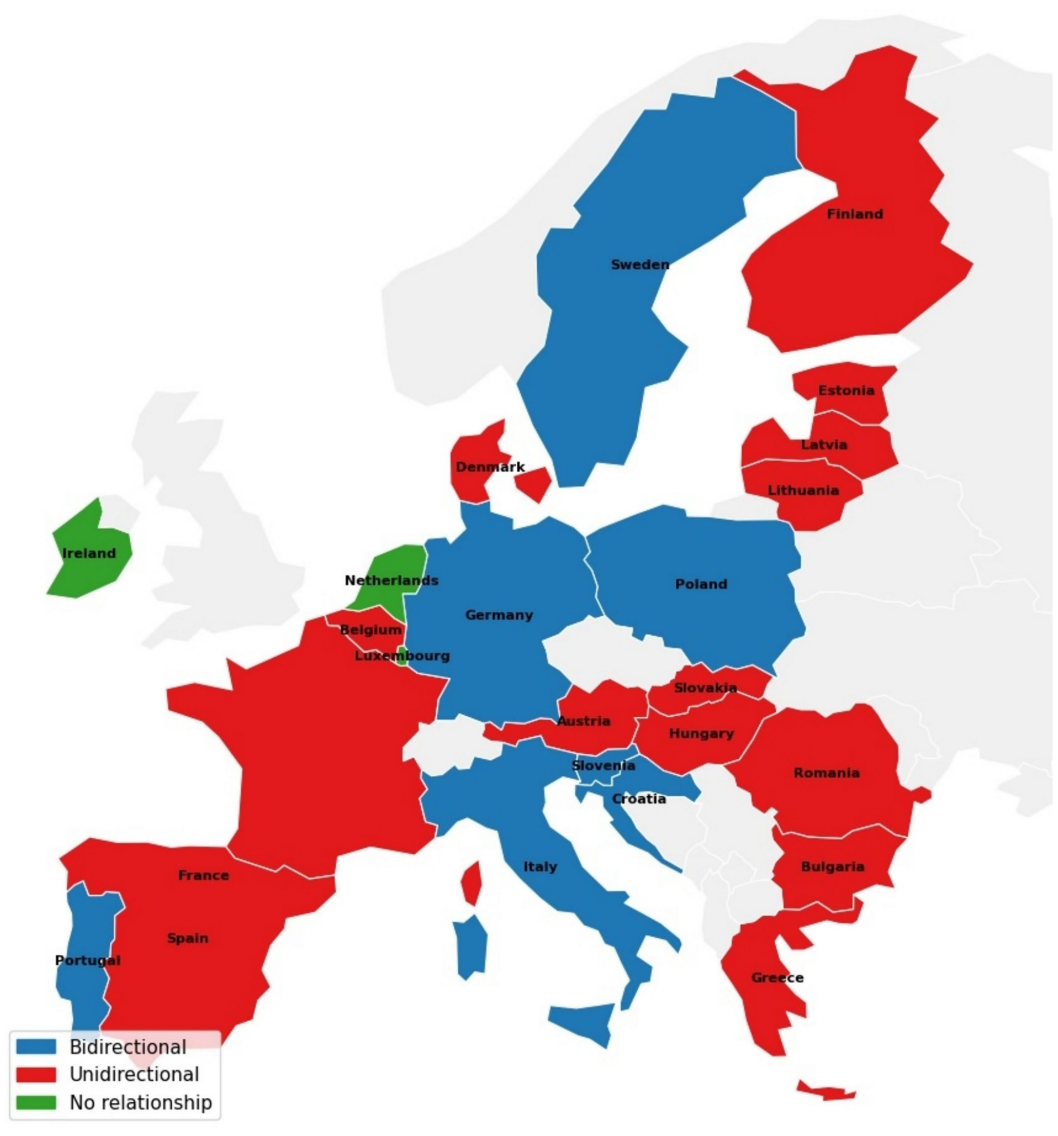
Granger causality patterns across EU member states. Countries are classified based on the country-level results reported in Tables 3–5. Bidirectional: significant Granger causality in both directions for at least one variable pair. Unidirectional: significant Granger causality in at least one direction for at least one variable pair, but no bidirectional relationship for any pair. No relationship: no statistically significant Granger causality in any direction for any variable pair. Significance is assessed at the 10% level or below.

Figure 2 provides a visual summary of the Granger causality patterns across EU member states, aggregating the country-level results from Tables 3–5.

The classification reveals that EU countries do not cluster into a small number of common patterns; instead, the distribution is highly fragmented, with 21 distinct causality profiles across 27 countries (Table 8). Visual inspection of Figure 2 suggests no obvious regional clustering, bidirectional, unidirectional, and decoupled countries appear distributed across Northern, Southern, Eastern, and Western Europe, suggesting that the heterogeneity is not driven by broad regional patterns.

**Table 8:** Country typology of Granger-causality patterns across the CO₂–migration–health expenditure nexus.

Profile	CO₂ ↔ MIGR	HEXP ↔ MIGR	HEXP ↔ CO₂	Countries	n
1	None	MIGR → HEXP	CO₂ → HEXP	Austria	1
2	None	HEXP → MIGR	None	Belgium	1
3	None	MIGR → HEXP	None	Bulgaria; Spain	2
4	MIGR → CO₂	None	Bidirectional	Croatia	1
5	MIGR → CO₂	MIGR → HEXP	None	Cyprus	1
6	None	None	HEXP → CO₂	Czechia	1
7	MIGR → CO₂	None	None	Denmark	1
8	CO₂ → MIGR	HEXP → MIGR	HEXP → CO₂	Estonia	1
9	None	HEXP → MIGR	HEXP → CO₂	Finland	1
10	CO₂ → MIGR	HEXP → MIGR	CO₂ → HEXP	France	1
11	None	None	Bidirectional	Germany; Poland	2
12	CO₂ → MIGR	None	None	Greece; Latvia; Romania	3
13	MIGR → CO₂	HEXP → MIGR	CO₂ → HEXP	Hungary	1
14	None	None	None	Ireland; Luxembourg; Netherlands	3
15	Bidirectional	MIGR → HEXP	None	Italy	1
16	CO₂ → MIGR	None	CO₂ → HEXP	Lithuania	1
17	MIGR → CO₂	HEXP → MIGR	HEXP → CO₂	Malta	1
18	None	Bidirectional	None	Portugal	1
19	CO₂ → MIGR	HEXP → MIGR	None	Slovak Republic	1
20	None	Bidirectional	HEXP → CO₂	Slovenia	1
21	CO₂ → MIGR	MIGR → HEXP	Bidirectional	Sweden	1

Although the 21 profiles are individually distinct, several broader groupings emerge. Seven countries, Italy, Germany, Sweden, Croatia, Poland, Portugal, and Slovenia, exhibit at least one bidirectional variable pair, suggesting feedback dynamics. Four countries, Lithuania, Romania, Latvia, and Greece, show CO₂ as the dominant source variable, predicting migration and/or health expenditure without significant reverse linkages. Three countries, Belgium, Czechia, and Finland, show HEXP as the dominant source, while four, Denmark, Cyprus, Spain, and Bulgaria, show MIGR as the dominant source. Austria, Estonia, France, Hungary, and Malta exhibit multi-linkage profiles in which more than one source variable is active across equations, making them less easily classified under a single dominant driver. Slovakia displays a migration-recipient profile in which both CO₂ and HEXP predict MIGR. Ireland, Luxembourg, and the Netherlands show no significant linkages in any direction. These broader groupings are interpreted as regime types in Section 5, where they are linked to the structural mechanisms underlying each pattern. In total, seven countries exhibit at least one bidirectional variable pair, 17 show at least one unidirectional linkage without bidirectionality, and three, Ireland, Luxembourg, and the Netherlands, show no significant relationship in any direction.

Although Table 8 identifies 21 distinct country profiles, these patterns can be interpreted through a smaller set of structural moderators, including country size, energy mix, health-system design, and migration exposure, that condition whether the nexus emerges as bidirectional, unidirectional, or absent. These moderators offer plausible explanations for why small or infrastructure-constrained states such as Malta display migration-driven emissions patterns, why Portugal and Slovenia exhibit feedback between migration and health expenditure within broad-coverage systems, and why Germany, Poland, Sweden, and Croatia show bidirectional links between health expenditure and CO₂ in settings where both pollution-related disease burden and healthcare-sector emissions are substantial. The discussion that follows develops these mechanisms using representative country cases.

## 5 Discussion

The panel-level Fisher test statistics confirm bidirectional Granger causality for all three variable pairs, CO₂ and net migration (*λ* = 107.156 and 104.003, both *p* < 0.01), health expenditure and net migration (λ = 90.565 and 92.942, both *p* < 0.01), and health expenditure and CO₂ (λ = 115.396 and 117.682, both *p* < 0.01).

These panel-level results indicate that, across the EU-27 as a whole, each of the three variables contains statistically significant predictive information for the other two, suggesting that CO₂ emissions, health expenditure, and net migration are not independent policy domains, the pairwise Granger-causality results suggest that changes in any one variable carry predictive implications for the others.

### 5.1 CO₂ and migration

Consistent with the panel-level bidirectional result reported above, the country-level patterns for CO₂ and MIGR reveal substantial heterogeneity. At the country level, CO₂ significantly predicts migration in nine member states, migration significantly predicts CO₂ in six, and Italy is the only country exhibiting bidirectionality. The results for CO₂ and Migration suggest that, in some member states, environmental conditions may help predict migration dynamics, whereas in others net migration may be associated with later changes in emissions through shifts in population pressure, energy demand, and consumption patterns. This finding is consistent with literature suggesting that changing lifestyles and consumption patterns associated with migration may contribute to increased CO₂ emissions. CO₂ patterns may change as people migrate from rural to urban areas; however, evidence from outside the EU context suggests this relationship is not straightforward. For instance, Komatsu et al. (2) found that rural-to-urban migrants in Hanoi, though examining internal rather than international migration, consumed less energy and emitted less CO₂ than nonmigrants, which the authors attributed to differences in appliance ownership and consumption habits. According to the network analysis of Li et al. (69), approximately half of the carbon footprint flows embodied in international migration are directed from developing to developed countries. More broadly, the global literature suggests that income inequality drives a large portion of migration flows towards developed countries (70), where migrants’ increased income may lead to higher consumption of electrical appliances and energy, potentially raising carbon emissions. At the same time, population growth in urban areas may accelerate infrastructure transitions from coal to lower-carbon fuels such as natural gas and renewable energy, partially offsetting these effects. The Granger-causal relationship between CO₂ and MIGR may partly reflect differences in environmental quality across countries. Although this study uses CO₂ as the environmental indicator, the broader literature on local air pollutants, including PM2.5, NO₂, and SO₂, points to similar but context-dependent mechanisms linking environmental quality to migration patterns (6, 21).

The heterogeneity in the CO₂–MIGR relationship is consistent with cross-country differences in industrial geography, country size, and the carbon intensity of infrastructure. Italy’s bidirectional result (CO₂ → MIGR: Wald = 15.020, *p* = 0.002; MIGR → CO₂: Wald = 10.035, *p* = 0.018) is plausibly linked to the spatial overlap between migrant settlement and highly industrialised, polluted northern regions. Immigrants concentrate in the Po Valley, which produces roughly 40% of national Gross domestic product (GDP) (71), and Italy’s energy mix remained approximately 78% fossil fuels in 2021 (72), so that population growth occurs within a still carbon-intensive system, a structural context consistent with the MIGR → CO₂ predictive direction. The Po Valley is also among Europe’s worst air pollution hotspots, Italy recorded the highest number of premature deaths from NO₂ in the EU in 2021 (73), and these environmental conditions are consistent with the CO₂ → MIGR direction, in which past emissions contain predictive information for subsequent migration patterns.

The unidirectional MIGR → CO₂ results in Malta (Wald = 16.399, *p* = 0.001), Denmark (Wald = 10.533, *p* = 0.005), and Croatia (Wald = 20.105, *p* < 0.001) are more consistent with small-country or infrastructure-constrained effects. Malta’s population grew 41% between 2004 and 2024, driven almost entirely by immigration (74), in an economy of approximately 563,000 people with virtually no domestic energy resources and historically oil-dependent power generation. Denmark’s result is notable because, despite world-leading wind energy penetration providing 54% of electricity, its remaining emission-intensive sectors, transport and heating, are precisely those most affected by population growth (75). Croatia’s MIGR → CO₂ result is consistent with the labour migration associated with its large tourism sector, 17.8 million tourist arrivals in 2023 against a resident population of 3.8 million (76), which attracts service-sector workers captured in net migration while placing substantial seasonal energy demand on carbon-intensive infrastructure. This pattern is consistent with the IPAT identity (17): in small or infrastructure-constrained states, the population component of environmental impact is amplified by high per-capita consumption intensity and limited technological offsetting.

These aggregate-level findings are consistent with Liang et al. (18), whose global estimates show that emissions embodied in international migration reached 2.9 Gt by 2015, a non-trivial share that rises disproportionately in small, carbon-intensive destination economies. The opposite unidirectional pattern, CO₂ → MIGR, observed in Estonia (Wald = 5.219, *p* = 0.022), France (Wald = 5.623, *p* = 0.060), Lithuania (Wald = 8.832, *p* = 0.032), and Romania (Wald = 19.058, *p* < 0.001), is more consistent with pollution-intensive industrial legacies or environmental inequality. Estonia’s oil shale industry historically accounted for the large majority of national CO₂ emissions and over 80% of electricity generation (77), concentrating environmental damage in Ida-Viru County, whose population declined by roughly one-third between the 1990s and 2020 (78), though this subnational decline may partly reflect internal relocation rather than international emigration. Romania experienced one of the European Union (EU)‘s largest emigration waves, with an estimated 3.6 million nationals abroad (79), against a backdrop of high environmental health costs, per-capita health costs from air pollution were the highest in the EU at €1,810 per year (80) though the emigration is widely attributed to economic rather than environmental factors. Lithuania lost more than one-fifth of its population since 1989, with approximately 80% of the decline attributable to emigration (81, 82). While some of this outflow originated in regions with Soviet-era industrial legacies, the emigration is primarily attributed to economic factors, and the subnational pollution concentration does not map directly onto the national net migration variable used in this study. France’s CO₂ → MIGR result is harder to interpret. Although Padilla et al. (83) found that immigrant communities in Paris and Lille are disproportionately located in higher-pollution areas, this documents exposure inequality among settled immigrants rather than evidence that pollution drives migration flows. The predictive relationship may instead reflect broader structural co-movement between France’s industrial emissions trajectory and its labour migration patterns. Overall, the CO₂–MIGR results suggest that migration–emissions linkages are strongest where population movements interact with either highly carbon-intensive infrastructure or environmentally degraded regions, with the specific direction determined by whether pollution acts as a push factor or population growth acts as an emissions amplifier.

The CO₂ → MIGR direction in these countries aligns with the push–pull framework (16), in which environmental degradation functions as a push factor at origin, complementing the economic and political drivers traditionally emphasised in migration research. The broader empirical literature supports this heterogeneity: Wang et al. (6) found that increased levels of air pollutant emissions significantly deter migration, while Squalli (21) found that US states with higher proportions of foreign-born residents are associated with lower NO₂ and SO₂ emissions, illustrating how the direction and strength of the migration–emissions relationship varies across institutional and geographic contexts. By contrast, Gao et al. (84) found that environmental health factors, including air quality, have not yet become significant drivers of intercity migration in China, where socioeconomic incentives remain dominant. While the comparison is limited by differences in migration type (internal vs. international) and institutional context, it is consistent with the possibility that environmental factors play a larger role in migration decisions where basic economic needs are more fully met.

### 5.2 HEXP and migration

The country-level results for HEXP and MIGR likewise show heterogeneous patterns behind the panel-level bidirectional finding. At the country level, HEXP predicts migration in nine member states, migration predicts HEXP in eight, and Portugal and Slovenia are the only countries exhibiting bidirectionality. These findings suggest that migration and health expenditure are linked through multiple channels, including demographic composition, health-system design, and labour-market conditions, that operate differently across member states. Table 4 findings are consistent with the existing evidence showing that migration dynamics and health expenditure can be linked through demographic composition, health status, access barriers, and host-country institutional arrangements.

One plausible mechanism is nonetheless suggested by the literature. It should be noted that several of the studies cited below examine specific immigrant subgroups or individual-level healthcare expenditures rather than aggregate net migration and per capita HEXP; as with the measurement differences flagged in Section 2, these studies provide adjacent rather than directly comparable evidence. Although MIGR captures net migration rather than immigrant stock or share, Bettin & Sacchi (3) found that a higher immigrant share in the resident population is associated with lower per capita health expenditure, attributing this to the younger and healthier demographic profile of migrant populations. This pattern, known as the healthy immigrant effect (45), may help explain why net migration contains predictive information for health expenditure in several EU countries. However, this health advantage is not uniform across all immigrant categories; Lu & Ng (47) found that while the effect persists among family-class immigrants, it is substantially weaker among refugees, a group that may require additional health monitoring and thus exert greater pressure on healthcare expenditures over time. Evidence from the US suggests that undocumented migrants may avoid seeking healthcare due to fear of immigration enforcement (85), which in that context has been associated with lower healthcare utilisation and costs among this group (86).

Over the longer term, however, the literature on migrant health trajectories, which relates to accumulated immigrant stocks rather than the net migration flows captured by MIGR, suggests that healthcare systems may face increasing pressure as settled migrant populations age and their health profiles converge with those of native-born populations. For instance, McDonald & Kennedy (87) found that the prevalence of chronic health issues among long-term immigrants in Canada was similar to that of the Canadian-born population after approximately 20 years. However, limited research examines the relationship between HEXP and MIGR directly. Migration scholarship typically emphasises economic and political drivers, while healthcare expenditure is rarely considered a primary determinant of migration flows. Instead, the broader migration literature identifies factors such as low wages, unemployment, violence, and political instability as key drivers (49). Within the EU specifically, Fassmann (50) points out that two-thirds of migrants move to Europe for family reunification and employment reasons. While the Granger-causality finding that HEXP predicts MIGR does not imply that migrants consciously respond to national health expenditure levels, it may reflect broader structural factors, such as economic development and public-service infrastructure, that simultaneously shape both health spending and destination-country attractiveness.

The country-level patterns in Table 4 are consistent with differences in health-system design, migrant coverage arrangements, and demographic or labour-market conditions. Portugal (HEXP → MIGR: Wald = 6.615, *p* = 0.085; MIGR → HEXP: Wald = 7.824, *p* = 0.050) and Slovenia (HEXP → MIGR: Wald = 7.578, *p* = 0.056; MIGR → HEXP: Wald = 12.951, *p* = 0.005), the two countries showing bidirectional Granger causality, are both small or medium-sized systems in which immigration can have noticeable effects on service demand while health-system accessibility may also form part of broader destination attractiveness. Portugal’s universal NHS-type system, with health expenditure at 10.2% of GDP (88), provides an institutional context through which migration and health spending interact; during COVID-19, Portugal further extended access by temporarily regularising undocumented migrants’ healthcare entitlements (89). Slovenia operates a Bismarck-type compulsory health insurance system with near-universal statutory coverage, covering more than 99% of permanent residents, and as a small country of approximately 2.1 million people, even modest immigration flows from the Western Balkans, 79% of 2021 immigrants came from outside the EU (90), have proportionally significant impacts on health system demand. These bidirectional results are broadly consistent with adjacent evidence from Bettin & Sacchi (3), who, using immigrant share rather than net migration, found that a 1 percentage point increase in Italy’s immigrant population reduces public health expenditure per capita by approximately 3.8%, and with Rana et al. (15), who documented significant immigration effects on both public and out-of-pocket health expenditure across OECD countries.

The unidirectional MIGR → HEXP results in Austria (Wald = 8.184, *p* = 0.042), Sweden (Wald = 20.038, *p* < 0.001), Spain (Wald = 4.872, *p* = 0.027), and Bulgaria (Wald = 12.458, *p* = 0.002) appear to reflect different mechanisms. Austria and Sweden both experienced dramatic migration shocks during the 2015 refugee crisis: Austria received 88,340 asylum applications (91), representing over 1% of its population, while Sweden received 162,877, the highest per capita in the EU among destination countries (92). Both countries provide comprehensive healthcare access to asylum seekers. Sweden’s total migration-related costs reached approximately €6 billion (1.35% of GDP) in 2015 (93), of which healthcare was one component, illustrating the broader fiscal pressure that large migration inflows place on public services, including but not limited to health expenditure. Research using Austrian administrative data found that refugees’ health expenditures were initially higher than natives’, reflecting inferior health status upon arrival, but converged downward over time, particularly after a positive asylum decision (94).

This convergence pattern is consistent with an initial catch-up in healthcare needs followed by stabilisation, though the supporting evidence, though the supporting evidence, including McDonald & Kennedy (87), who found convergence in chronic health conditions among long-term immigrants in Canada after approximately 20 years, examines immigrant stocks rather than the net migration flows captured by MIGR. Spain’s MIGR → HEXP result coincides with the 2000s immigration boom, during which the foreign-born population surged from 1.66 million (2000) to 6.28 million (2010). Although the Granger test identifies predictive direction rather than the sign of the expenditure effect, adjacent evidence suggests that migrant healthcare utilisation in Spain converges with that of natives after approximately 15 years (95), which is consistent with a gradual shift in the composition of healthcare demand. Bulgaria presents a different pattern: MIGR → HEXP may partly reflect broader net out-migration, one component of which is the substantial emigration of healthcare workers, approximately 500 doctors per year since EU accession in 2007 (96), compounded by chronic nursing shortages that have left Bulgaria with one of the lowest nurse densities in the EU (97). While the MIGR variable captures aggregate net migration rather than healthcare-worker flows specifically, the loss of medical personnel is a plausible channel through which net out-migration could be associated with rising per-patient cost.

The HEXP → MIGR results in France (Wald = 13.041, *p* = 0.005), Belgium (Wald = 5.358, *p* = 0.069), and Hungary (Wald = 9.284, *p* = 0.026) indicate that the same causal direction can reflect different substantive processes across countries. France’s Aide Médicale d’État (AME) provides comprehensive health coverage to undocumented migrants, with over 466,000 beneficiaries by 2023; although a 2023 bipartisan report concluded that AME does not appear to be a primary attractive factor for prospective immigrants (98), the broader universal coverage system creates an institutional environment that may facilitate migrant settlement. Belgium combines high-quality healthcare (11.0% of GDP) with Brussels’ unique status as EU and NATO headquarters, which may contribute to destination attractiveness. Hungary’s pattern operates in the opposite direction: health expenditure of only 6.5% of GDP, well below the OECD average of 9.3% (88) and life expectancy 4.4 years below the OECD average (88), contribute to emigration of healthcare professionals and working-age populations to Germany, Austria, and the UK. This suggests that migration–health expenditure linkages depend less on migration per se than on how migrant inflows and outflows are filtered through national health institutions. These patterns are consistent with the push–pull framework applied to health systems: universal coverage and high-quality services may function as pull factors in France and Belgium, while underfunded systems function as push factors in Hungary. Grossman (27) health capital model provides a micro-foundation for this direction: immigration alters the demographic composition and health capital distribution of destination populations, generating predictable changes in aggregate health expenditure. Adjacent individual-level evidence is consistent with this mechanism: Goldman et al. (99) found that immigrants in the United States incur lower healthcare costs than natives, and Flavin et al. (41) confirmed that immigrant populations generally use less healthcare than comparable native-born populations, though these studies examine immigrant subgroups rather than aggregate net migration, though both studies note that utilisation patterns shift over time as immigrant populations age and settle.

### 5.3 HEXP and CO₂

Turning to HEXP and CO₂, the country-level decomposition again reveals diverse causal patterns. At the country level, HEXP predicts CO₂ in nine member states, CO₂ predicts HEXP in eight, and Germany, Sweden, Croatia, and Poland exhibit bidirectionality. These results suggest that the health–emissions relationship operates through two channels: pollution-driven health costs on one side, and the carbon footprint of healthcare delivery on the other. It should be noted that because HEXP is measured in current US dollars, year-to-year variation reflects not only real changes in healthcare activity but also price movements and exchange-rate effects; the structural interpretations that follow assume that HEXP variation is at least partly driven by real resource use, but this cannot be verified with the aggregate expenditure measure used in this study. Table 5 findings suggest that the relationship between health expenditure and emissions is heterogeneous across EU member states. On the one hand, higher emissions may be associated with greater health-system pressures and expenditure needs; on the other hand, expansion of health services may itself be energy-intensive and therefore linked to higher emissions.

Previous panel evidence suggests that carbon emissions and healthcare expenditure may exhibit bidirectional relationships, particularly in multi-country settings (31). CO₂ emissions contribute to climate change, which the literature associates with a range of adverse health outcomes, including heat-related illness, waterborne diseases, and increased allergen exposure, that may in turn place upward pressure on healthcare costs (100–102). Additionally, although CO₂ itself is not a toxic pollutant, it shares common combustion sources with pollutants such as PM2.5 and NO₂. While not directly measuring CO₂, Jerrett et al. (22) found that higher levels of such ambient pollution are associated with higher HEXP per person, suggesting a related but distinct channel through which fossil fuel combustion may affect health spending. Likewise, expansion of healthcare systems may contribute to higher CO₂ emissions through an indirect feedback channel, as population growth, including migration-driven growth, increases healthcare demand and may lead to the construction of larger facilities and greater energy consumption.

Reducing CO₂ emissions and related health-expenditure pressures may benefit from well-coordinated strategies. Policies that reduce CO₂ emissions may help ease pollution-related health care costs, as evidence from US states shows that higher per capita emissions are associated with higher per capita health care expenditures (20).

The HEXP–CO₂ relationship likewise appears to be conditioned by differences in energy mix, pollution burden, and the emissions profile of healthcare systems themselves. Germany (HEXP → CO₂: Wald = 5.996, *p* = 0.050; CO₂ → HEXP: Wald = 9.144, *p* = 0.010), Poland (Wald = 5.730, *p* = 0.057; Wald = 8.332, *p* = 0.016), Sweden (Wald = 5.311, *p* = 0.070; Wald = 8.060, *p* = 0.018), and Croatia (Wald = 8.376, *p* = 0.039; Wald = 19.483, *p* < 0.001) exhibit bidirectional Granger causality. While the Granger test identifies predictive direction rather than specific mechanisms, the bidirectional pattern in these countries is consistent with a plausible feedback loop in which pollution-related disease burdens drive health costs while the energy and supply-chain demands of healthcare delivery contribute to emissions. Germany’s healthcare sector accounted for approximately 5–6.7% of national GHG emissions (103), and total energy supply remained predominantly fossil-based (104). Poland offers a particularly clear case of a coal-intensive power system: coal accounted for about 70.7% of electricity generation in 2022, while total energy supply remained overwhelmingly fossil-based IEA (104), Poland recorded 25,268 premature deaths from PM2.5 in 2023 and one of the highest PM2.5-attributable mortality rates in the EU (105). In both cases, CO₂ may predict health expenditure through pollution-related disease burdens, while health expenditure may predict CO₂ because expanding care provision, hospital activity, and medical supply chains are themselves emissions-intensive. The CO₂ → HEXP direction is consistent with Apergis et al. (20), who found that higher per capita emissions across US states are associated with higher per capita health care expenditures, and with Zaidi & Saidi (8), who reported similar linkages in Sub-Saharan Africa using panel ARDL. Wang et al. (32), using bootstrap ARDL across 18 OECD countries, similarly found bidirectional causality between health expenditure and CO₂ in only two countries, with unidirectional patterns predominating, a pattern of country-level heterogeneity consistent with the present findings.

Sweden and Croatia illustrate that bidirectionality need not require the same underlying national profile. Sweden’s health expenditure represents 11.2% of GDP (78), and although electricity generation is relatively clean, supply-chain emissions dominate, consistent with the EU-wide pattern where they account for approximately 71% of health sector emissions (106). More broadly, Pichler et al. (29) estimated that OECD health systems are responsible for 4.4% of global GHG emissions, highlighting the sector’s substantial carbon footprint even in high-income countries. Croatia’s bidirectional result reflects significant PM2.5 pollution concentrated in specific regions, notably Brodsko-posavska županija, which had one of the highest PM2.5-attributable death rates in the EU in 2023 (105), alongside health expenditure growth of 108.9% per inhabitant between 2014 and 2023 (78).

The unidirectional HEXP → CO₂ results in Estonia (Wald = 16.396, *p* < 0.001), Finland (Wald = 4.909, *p* = 0.027), and Malta (Wald = 24.973, *p* < 0.001) share a common structural feature: small economies where healthcare infrastructure expansion materially affects national energy demand. Estonia’s continued dependence on oil shale (56% of total energy supply in 2023) (107) suggests that healthcare expansion carries an amplified carbon intensity. Finland’s sparse population and vast distances mean healthcare delivery requires significant transport infrastructure, and the country exhibits absolute coupling of emissions and energy consumption (108), Malta’s constrained micro-island economy implies that healthcare modernisation would place additional demand on an energy system with very low renewable penetration.

The CO₂ → HEXP results in Austria (Wald = 7.179, *p* = 0.066), France (Wald = 7.057, *p* = 0.029), Hungary (Wald = 12.356, *p* = 0.006), and Lithuania (Wald = 9.666, *p* = 0.002) reflect pollution-driven health costs through different channels. Hungary presents the strongest evidence: global health costs per capita from ambient PM2.5 combustion sources are among the highest worldwide at $660 (109), with 6,767 premature deaths from PM2.5 in 2023 (105). Austria’s pathway is driven by its role as an Alpine transit corridor, where the transport sector accounts for approximately 25% of total CO₂ emissions and valley topography traps pollutants. France demonstrates this direction despite nuclear energy providing approximately 70% of electricity, because transport and industrial sectors remain significant emission sources; Mink (110) estimated short-term healthcare costs of PM2.5 exposure in France to be 2–6 times larger than prior estimates. Lithuania shows the CO₂ → HEXP direction through rapidly growing transport emissions, which increased 43.4% from 2010 to 2019, the largest increase among all EU countries (111). These findings reinforce that the health–environment nexus is mediated not only by ambient pollution but also by how carbon-intensive the health sector itself remains. The CO₂ → HEXP direction is consistent with (27) depreciation mechanism, pollution accelerates health capital loss, requiring greater health expenditure, amplified in countries with high ambient pollution exposure such as Hungary and Poland. Chaabouni et al. (7) found similar bidirectional dynamics between CO₂ and health expenditure, while Slathia et al. (31) reported bidirectional linkages in Asian countries, suggesting that the health–emissions feedback is not unique to the EU but may be a general feature of economies where healthcare expansion and fossil-fuel dependence coexist.

These structural interpretations across all three variable pairs point to three dominant conditioning mechanisms. First, country size mediates the migration–emissions relationship: in small states (Malta, Estonia, Denmark), population changes have outsized per-capita impacts on emissions, while in large, geographically diverse states (Italy, France), the relationship operates through spatial concentration of migrants in industrial or polluted areas. Second, health-system design determines the migration–health expenditure pathway: universal systems with explicit migrant coverage create institutional channels through which migration and health spending interact, while systems experiencing medical brain drain (Hungary, Bulgaria) show the reverse, poor health-system conditions associated with outward mobility. Third, energy mix composition is the critical moderator of the health–CO₂ nexus: coal-dependent economies (Poland, Estonia) show stronger pollution-to-health-cost transmission, while countries with large healthcare sectors in still partly fossil-fuel-dependent economies (Germany, Croatia) show bidirectional feedbacks between health sector emissions and pollution-driven health demand.

These conditioning mechanisms suggest that EU member states can be broadly characterised as operating within one of four regime types in the CO₂–health expenditure–migration nexus. First, integrated nexus countries, Italy, Germany, Sweden, Croatia, Poland, Portugal, and Slovenia, exhibit bidirectional feedback in at least one variable pair, reflecting settings where migration, emissions, and health expenditure are structurally intertwined through industrial geography, healthcare-sector emissions, or universal health-system design. Second, environment-sensitive migration regimes, Estonia, France, Lithuania, and Romania, show CO₂ as the dominant source variable, consistent with pollution-intensive industrial legacies or environmental inequality that may shape population movements without a comparably strong reverse channel. Third, demographic-pressure-driven systems, Malta, Denmark, Croatia, Cyprus, Spain, and Bulgaria, show migration as the dominant source variable, consistent with settings where population inflows or outflows place measurable pressure on emissions or health expenditure through infrastructure demand or workforce depletion. Fourth, structurally decoupled systems, Ireland, Luxembourg, and the Netherlands, show no significant Granger-causal linkages in any direction, though non-significance may partly reflect the limited statistical power of the country-level tests given the short time dimension and the smoothed migration series rather than genuine independence among the variables. Austria, Estonia, France, Hungary, and Malta exhibit multi-linkage profiles that span more than one regime type, while Belgium, Czechia, Finland, Greece, Latvia, and Slovakia show simpler unidirectional patterns, predominantly HEXP-dominant or CO₂-dominant, that do not map neatly onto the four regime categories. These residual cases underscore that the typology captures the most analytically interpretable configurations rather than providing an exhaustive classification of all 27 member states. The Granger-causality patterns which classifies countries as bidirectional, unidirectional, or showing no significant relationship, provide the empirical basis from which these regime types are derived.

### 5.4 CS-ARDL robustness

The CS-ARDL results complement the Granger causality findings by providing evidence on the long-run magnitude and sign of the relationships in the CO₂ equation. The significant negative long-run coefficient of health expenditure on CO₂ is consistent with the possibility that health-system investments, including technological upgrading and efficiency improvements in healthcare delivery, may be associated with lower emissions over time, a pattern that aligns with evidence on the carbon footprint of healthcare systems in high-income countries (29). The positive long-run coefficient of net migration on CO₂ is consistent with the IPAT framework (17) and with the Granger causality results showing that migration predicts CO₂ in several member states: population growth in destination regions, combined with shifts in consumption and energy demand, may contribute to higher aggregate emissions. The limited short-run effects and the significant error correction term suggest that these relationships operate primarily through gradual, structural channels rather than immediate shocks, a finding that supports the case for long-term, integrated policy planning rather than reactive interventions. It should be noted that this robustness analysis estimates only the CO₂ equation; the long-run magnitudes of the reciprocal effects (i.e., of CO₂ on health expenditure and migration) remain to be estimated in future work.

### 5.5 Policy implications

The policy implications are informed by the regime typology and structural mechanisms identified above, though they should be read as indicative directions rather than prescriptions, given that the underlying evidence rests on predictive relationships and an interpretive classification. Because several countries span more than one regime type, the policy priorities below are organised by the dominant pattern in each country rather than implying exclusive membership in a single category.

The spatial distribution of the underlying Granger-causality patterns does not appear to cluster by geographic region, reinforcing the need for country-specific rather than regionally uniform policy coordination. In countries classified here as demographic-pressure-driven systems, such as Malta, Denmark, and Croatia, where migration is the dominant source variable, the priority is to decarbonise the infrastructure through which population growth is absorbed. In Malta, where the discussion documents virtually no domestic energy resources and historically oil-dependent power generation, this implies accelerating renewable energy deployment and reducing transport-sector fossil fuel dependence, sectors where even modest population inflows have outsized emissions effects. In Denmark, where transport and heating remain emission-intensive despite clean electricity generation, migrant-integration strategies should be coupled with building retrofits and public transport expansion. Croatia’s tourism-driven population surges reinforce the case for seasonal energy-demand management alongside labour-immigration planning. In countries classified here as environment-sensitive migration regimes, such as Estonia, Lithuania, and Romania, where CO₂ is the dominant source, pollution reduction and environmental remediation may yield benefits beyond climate policy by reducing environmental push factors and place-based inequality.

Estonia’s experience with the Just Transition Fund in Ida-Viru County, a region whose population declined by roughly one-third between the 1990s and 2020 (78), illustrates how targeted regional diversification can address environmental degradation while supporting economic resilience in depopulating areas (112). Lithuania’s rapidly growing transport emissions (111), the largest increase among all EU countries between 2010 and 2019, suggest that transport decarbonisation is a priority not only for climate targets but also for reducing pollution-driven health costs in a region where Yin et al. (109) identified substantial disparities between combustion-source health costs and PM2.5 concentrations. In countries classified here as integrated nexus cases, such as Portugal, Slovenia, as well as multi-linkage cases such as Austria and Sweden, where migration and health expenditure are linked, policy should focus on migrant-inclusive primary care, language-accessible services, and workforce planning (25, 41, 43, 113). The Austrian convergence pattern implies that early investment in preventive care and primary access can moderate long-term expenditure growth, making inclusive coverage. Portugal’s experience with extending healthcare access during COVID-19 (89) and Spain’s evidence that migrant healthcare utilisation converges with natives after approximately 15 years (95) reinforce that inclusive coverage is not only an equity measure but a fiscal planning tool. In Bulgaria, where health-workforce depletion has weakened care capacity (96, 97), retention policies, including the salary reforms already attempted, need to be paired with training-pipeline investments to break the emigration–underservice cycle.

Among integrated nexus countries where the bidirectional linkage involves health expenditure and CO₂, such as Germany, Poland, Sweden, and Croatia, the latter also classified above as a demographic-pressure case, illustrating the non-exclusive nature of the typology, governments should pair pollution-control policies with decarbonisation of healthcare operations (20). Given the healthcare sector’s substantial emissions footprint, dominated by pharmaceutical production, medical equipment, and logistics rather than direct hospital energy use (106), procurement reform, supply-chain standards, and circular-economy principles in medical waste management may be more effective than facility-level energy efficiency alone. In Poland, where coal dependence and PM2.5-attributable mortality remain among the highest in the EU (105), accelerating the coal phase-out has direct co-benefits for both climate targets and health expenditure containment.

For the structurally decoupled countries identified in Section 4.3, the absence of significant linkages suggests less immediate pressure for cross-domain coordination, though this finding should be reassessed as longer time series become available. Building on the regime typology identified in Section 4.3, the policy implication is that a coordinated EU framework should allow regime-specific priorities rather than prescribing a single common policy package.

## 6 Conclusion

This study examined bidirectional Granger-causal relationships among CO₂ emissions, health expenditure, and net migration across all 27 EU member states over the period 2000–2020. At the panel level, all three variable pairs exhibit bidirectional Granger causality, while country-level patterns are heterogeneous.

Beneath this panel-level consensus, however, the country-level landscape is far more fragmented. Bidirectional feedback emerges in only seven member states, unidirectional linkages characterise 17 others, and three countries show no detectable relationship at all. The structural interpretation developed in the discussion suggests that this heterogeneity is consistent with differences in energy systems, health-sector organisation, and migration exposure, giving rise to four broad regime types that cut across geographic boundaries, though these moderators were not formally tested.

As the regime-specific patterns in Section 4.3 demonstrate, effective policy coordination must be tailored to each member state’s position within the nexus. Countries exhibiting demographic-pressure patterns require decarbonisation of the infrastructure absorbing population growth; those exhibiting environment-sensitive patterns benefit most from pollution remediation and place-based reinvestment; integrated nexus cases need migrant-inclusive healthcare planning and early prevention; and feedback cases call for simultaneous action on pollution control and healthcare-sector emissions. Where no significant linkages are detected, the case for cross-domain coordination is weaker, subject to the statistical power caveats noted in Section 4.3.

Several limitations should be acknowledged. First, the Emirmahmutoğlu and Köse (13) test establishes Granger-causal (predictive) relationships rather than structural causality; the findings indicate that past values of one variable contain statistically significant information for predicting another, but do not identify the underlying causal mechanisms or estimate the magnitude of the effects. Second, CO₂ emissions per capita serves as a macro-level environmental proxy and does not capture the full range of pollutants (e.g., PM2.5, NO₂) that may influence both health outcomes and migration decisions. Third, net migration aggregates heterogeneous population flows, including labour migrants, family reunification, and refugees, whose effects on health expenditure and emissions may differ substantially. Fourth, as noted in Section 2, HEXP captures system-level resource allocation and should not be interpreted as a measure of healthcare quality, access, or individual utilisation. Fifth, HEXP is denominated in current US dollars, so its time variation conflates real resource use with inflation and exchange-rate movements; purchasing-power-parity alternatives exist but were not used in this study. Sixth, the five-year interpolation of the net migration series, introduces artificial persistence that should be borne in mind when interpreting the annual Granger-causality results involving MIGR. Seventh, the pairwise specification does not control for potential confounders such as GDP per capita, urbanisation, or demographic structure. While this choice is motivated by degrees-of-freedom constraints, adding variables to the VAR system with T = 21 and *N* = 27 would risk overfitting— and while the Emirmahmutoğlu and Köse (13) framework treats all included variables as endogenous, the omission of additional variables may in principle affect Granger causality inference (54), and this should be borne in mind when interpreting the results. In particular, because each variable pair is tested in a separate bivariate VAR, the Granger-causality results for any given pair do not condition on the remaining variable; it is therefore possible that some pairwise linkages reflect indirect channels rather than direct predictive relationships. Eighth, the sample period ends in 2020, determined by the availability of the CO₂ emissions series, and therefore excludes post-COVID migration dynamics, including the 2022 Ukrainian refugee crisis and post-pandemic labour mobility shifts, that may have altered the migration–emissions–health expenditure nexus in ways not captured by the present analysis.

These limitations point to several directions for future research. Extending the framework to include disaggregated migration categories would allow for more precise identification of which population flows drive the observed relationships. Extending the sample period beyond 2020 as updated CO₂ data become available would allow assessment of whether post-COVID migration dynamics and the Ukrainian refugee crisis have altered the Granger-causal linkages identified here. Augmenting the VAR system with additional variables, such as GDP per capita, urbanisation, or demographic structure, in settings with longer time series would help assess the robustness of the identified Granger-causal linkages to omitted-variable concerns. Incorporating broader environmental indicators beyond CO₂ could provide a more comprehensive picture of the environment–health expenditure nexus. Extending the CS-ARDL analysis to the health expenditure and net migration equations would provide a more complete picture of the long-run reciprocal dynamics. Finally, examining how labour market dynamics and demographic structure condition these relationships at the country level would further strengthen the evidence base for integrated policy design.

## Data Availability

Publicly available datasets were analysed in this study. This data can be found at: CO₂ emissions (metric tons per capita): https://data.worldbank.org/indicator/EN.ATM.CO2E.PC. Current health expenditure per capita (current US$): https://data.worldbank.org/indicator/SH.XPD.CHEX.PC.CD; Net migration: https://data.worldbank.org/indicator/SM.POP.NETM.
